# Supplementary material for: Negotiating science funding: The interplay of merit, bias, and administrative discretion in grant allocation in Kazakhstan
Source: PLoS One. 2025 May 30;20(5):e0318875. doi: 10.1371/journal.pone.0318875 (PMC12124552; doi:10.1371/journal.pone.0318875)
Supplement: S1 Table — (DOCX) [file pone.0318875.s001.docx]

| metric | Culture | Agriculture | Science | Life | Security | Natural_rm | Energy |
| --- | --- | --- | --- | --- | --- | --- | --- |
| prev_success_rate | 0.16 | 0.18 | 0.29 | 0.25 | 0.12 | 0.07 | 0.22 |
| Rints_rate | 0.12 | 0.13 | 0.18 | 0.12 | 0.10 | 0.17 | 0.18 |
| Scopus_rate | 0.66 | 0.70 | 0.87 | 0.56 | 0.46 | 0.73 | 0.79 |
| Delisted_rate | 0.43 | 0.28 | 0.11 | 0.11 | 0.18 | 0.20 | 0.20 |
| Female_prop | 0.65 | 0.39 | 0.32 | 0.53 | 0.27 | 0.40 | 0.21 |
| hirsh0 | 0.73 | 0.61 | 0.31 | 0.63 | 0.79 | 0.50 | 0.40 |
| hirsh1 | 0.20 | 0.24 | 0.23 | 0.14 | 0.15 | 0.20 | 0.21 |
| hirsh2 | 0.04 | 0.10 | 0.14 | 0.08 | 0.04 | 0.12 | 0.13 |
| hirsh3+ | 0.02 | 0.06 | 0.32 | 0.15 | 0.02 | 0.17 | 0.25 |
| candidate_rate | 0.52 | 0.62 | 0.42 | 0.48 | 0.54 | 0.50 | 0.48 |
| doctor_rate | 0.39 | 0.33 | 0.40 | 0.41 | 0.31 | 0.39 | 0.34 |
| phd_rate | 0.09 | 0.05 | 0.18 | 0.12 | 0.15 | 0.11 | 0.18 |
