# Supplementary material for: Negotiating science funding: The interplay of merit, bias, and administrative discretion in grant allocation in Kazakhstan
Source: PLoS One. 2025 May 30;20(5):e0318875. doi: 10.1371/journal.pone.0318875 (PMC12124552; doi:10.1371/journal.pone.0318875)
Supplement: S2 Table — This table compares an ordinary OLS regression for Score variable (all predictors included) with a model with standard errors clustered by domains (column two), a model with random effects for PI, region, and domain, and an OLS regression with truncated residuals (tobit). (DOCX) [file pone.0318875.s002.docx]

|  | Full OLS | Clustered SE | Random Effects | Tobit |
| --- | --- | --- | --- | --- |
| (Intercept) | 22.66*** | 22.66*** | 22.40*** |  |
|  | (0.27) | (0.35) | (0.37) |  |
| sexFemale | -0.06 | -0.06 | -0.06 | -0.06 |
|  | (0.14) | (0.12) | (0.14) | (0.14) |
| regionАстана | 0.02 | 0.02 |  | 0.02 |
|  | (0.18) | (0.24) |  | (0.18) |
| regionШымкент | -1.34*** | -1.34 |  | -1.34*** |
|  | (0.31) | (0.86) |  | (0.31) |
| regionOther | -0.36+ | -0.36 |  | -0.36+ |
|  | (0.21) | (0.26) |  | (0.21) |
| domainAgriculture | 0.24 | 0.24* |  | 0.24 |
|  | (0.23) | (0.12) |  | (0.23) |
| domainScience | 0.17 | 0.17 |  | 0.17 |
|  | (0.25) | (0.24) |  | (0.25) |
| domainLife | -0.45+ | -0.45** |  | -0.45+ |
|  | (0.24) | (0.17) |  | (0.24) |
| domainSecurity | 0.39 | 0.39** |  | 0.39 |
|  | (0.44) | (0.14) |  | (0.44) |
| domainNatural_rm | 0.75*** | 0.75*** |  | 0.75*** |
|  | (0.20) | (0.11) |  | (0.20) |
| domainEnergy | 0.17 | 0.17 |  | 0.17 |
|  | (0.29) | (0.12) |  | (0.29) |
| rintsYes | 0.72*** | 0.72*** | 0.73*** | 0.72*** |
|  | (0.19) | (0.19) | (0.20) | (0.19) |
| scopusYes | 0.03 | 0.03 | 0.03 | 0.03 |
|  | (0.17) | (0.30) | (0.17) | (0.17) |
| hirsh | 0.26*** | 0.26*** | 0.26*** | 0.26*** |
|  | (0.03) | (0.04) | (0.03) | (0.03) |
| delistedYes | -0.64*** | -0.64 | -0.66*** | -0.64*** |
|  | (0.18) | (0.40) | (0.18) | (0.18) |
| win_2014Yes | 1.60*** | 1.60*** | 1.59*** | 1.60*** |
|  | (0.19) | (0.15) | (0.19) | (0.19) |
| degreeDoctor | 0.43** | 0.43** | 0.45** | 0.43** |
|  | (0.15) | (0.17) | (0.15) | (0.15) |
| degreePhD | 0.88*** | 0.88*** | 0.92*** | 0.88*** |
|  | (0.22) | (0.23) | (0.23) | (0.22) |
| inst_capWorks with | 0.76*** | 0.76* | 0.79*** | 0.76*** |
|  | (0.17) | (0.31) | (0.17) | (0.17) |
| inst_capMember | -0.68 | -0.68 | -0.51 | -0.68 |
|  | (0.53) | (0.55) | (0.54) | (0.53) |
| inst_cap(Missing) | -1.73** | -1.73+ | -1.67** | -1.73** |
|  | (0.63) | (1.04) | (0.64) | (0.63) |
| org_prestigeNational | -0.04 | -0.04 | -0.01 | -0.04 |
|  | (0.24) | (0.32) | (0.24) | (0.24) |
| org_prestigeInternational | 1.06 | 1.06 | 1.18+ | 1.06 |
|  | (0.67) | (0.82) | (0.68) | (0.67) |
| org_prestigeOther | 0.83*** | 0.83** | 0.83*** | 0.83*** |
|  | (0.20) | (0.31) | (0.20) | (0.20) |
| (Intercept) × 1 |  |  |  | 22.66*** |
|  |  |  |  | (0.27) |
| (Intercept) × 2 |  |  |  | 1.48*** |
|  |  |  |  | (0.01) |
| SD (Observations) |  |  | 3.80 |  |
| SD (Intercept pi_id) |  |  | 2.25 |  |
| SD (Intercept domain) |  |  | 0.34 |  |
| SD (Intercept region) |  |  | 0.57 |  |
| Num.Obs. | 4488 | 4488 | 4488 | 4488 |
| R2 | 0.107 |  |  |  |
| R2 Adj. | 0.103 |  |  |  |
| R2 Marg. |  |  | 0.086 |  |
| R2 Cond. |  |  | 0.339 |  |
| F | 23.327 |  |  |  |
| RMSE | 4.41 |  | 3.30 | 3.15 |
