# Supplementary material for: Negotiating science funding: The interplay of merit, bias, and administrative discretion in grant allocation in Kazakhstan
Source: PLoS One. 2025 May 30;20(5):e0318875. doi: 10.1371/journal.pone.0318875 (PMC12124552; doi:10.1371/journal.pone.0318875)
Supplement: S3 Table — This table presents two ordinal regression for Score variable (all predictors included). The first model takes all the possible values as ordered levels. The second model breaks original scores into 5 point intervals. (DOCX) [file pone.0318875.s003.docx]

|  | POLR | POLR, intervals |
| --- | --- | --- |
| sexFemale | -0.015 | 0.016 |
|  | (0.056) | (0.059) |
| regionАстана | 0.011 | 0.003 |
|  | (0.070) | (0.074) |
| regionШымкент | -0.487*** | -0.528*** |
|  | (0.122) | (0.128) |
| regionOther | -0.156+ | -0.187* |
|  | (0.082) | (0.087) |
| domainAgriculture | 0.055 | 0.085 |
|  | (0.089) | (0.095) |
| domainScience | 0.057 | 0.088 |
|  | (0.098) | (0.104) |
| domainLife | -0.183+ | -0.156 |
|  | (0.095) | (0.100) |
| domainSecurity | 0.125 | 0.113 |
|  | (0.178) | (0.186) |
| domainNatural_rm | 0.279*** | 0.332*** |
|  | (0.078) | (0.082) |
| domainEnergy | 0.090 | 0.116 |
|  | (0.115) | (0.121) |
| rintsYes | 0.255*** | 0.281*** |
|  | (0.076) | (0.081) |
| scopusYes | -0.034 | -0.032 |
|  | (0.068) | (0.071) |
| hirsh | 0.116*** | 0.116*** |
|  | (0.012) | (0.013) |
| delistedYes | -0.257*** | -0.221** |
|  | (0.069) | (0.073) |
| win_2014Yes | 0.662*** | 0.654*** |
|  | (0.074) | (0.079) |
| degreeDoctor | 0.152** | 0.158* |
|  | (0.058) | (0.061) |
| degreePhD | 0.322*** | 0.323*** |
|  | (0.088) | (0.093) |
| inst_capWorks with | 0.294*** | 0.275*** |
|  | (0.068) | (0.072) |
| inst_capMember | -0.329 | -0.271 |
|  | (0.202) | (0.216) |
| inst_cap(Missing) | -0.665* | -0.578* |
|  | (0.275) | (0.279) |
| org_prestigeNational | -0.020 | -0.041 |
|  | (0.095) | (0.100) |
| org_prestigeInternational | 0.366 | 0.167 |
|  | (0.275) | (0.291) |
| org_prestigeOther | 0.305*** | 0.272*** |
|  | (0.077) | (0.081) |
| Num.Obs. | 4488 | 4488 |
| AIC | 35855.5 | 11978.6 |
| BIC | 36528.5 | 12164.5 |
