# Supplementary material for: Negotiating science funding: The interplay of merit, bias, and administrative discretion in grant allocation in Kazakhstan
Source: PLoS One. 2025 May 30;20(5):e0318875. doi: 10.1371/journal.pone.0318875 (PMC12124552; doi:10.1371/journal.pone.0318875)
Supplement: S4 Table — This table presents four logistic regressions for Win. The first model is a logistic regression with robust standard errors. The second and third modes have clustered standard errors. The last model is a random effect (intercepts) model at the level of domains and regions. A model with a random intercept at the level of PI did not converge, nor did models with random slopes (at any level of grouping). (DOCX) [file pone.0318875.s004.docx]

|  | Robust SE | Clustered (PI) | Clustered (PI + Domain) | RE(Region + Domain) |
| --- | --- | --- | --- | --- |
| (Intercept) | -7.554*** | -7.554*** | -7.554*** | -7.585*** |
|  | (0.345) | (0.320) | (0.714) | (0.365) |
| score | 0.232*** | 0.232*** | 0.232*** | 0.232*** |
|  | (0.012) | (0.011) | (0.027) | (0.012) |
| hirsh | 0.019 | 0.019 | 0.019 | 0.019 |
|  | (0.017) | (0.018) | (0.016) | (0.017) |
| rintsYes | 0.121 | 0.121 | 0.121+ | 0.121 |
|  | (0.113) | (0.114) | (0.067) | (0.112) |
| scopusYes | 0.097 | 0.097 | 0.097 | 0.091 |
|  | (0.105) | (0.106) | (0.075) | (0.105) |
| delistedYes | -0.047 | -0.047 | -0.047 | -0.056 |
|  | (0.111) | (0.109) | (0.046) | (0.110) |
| win_2014Yes | 0.516*** | 0.516*** | 0.516*** | 0.519*** |
|  | (0.104) | (0.105) | (0.048) | (0.104) |
| degreeDoctor | 0.434*** | 0.434*** | 0.434*** | 0.444*** |
|  | (0.088) | (0.088) | (0.079) | (0.088) |
| degreePhD | -0.146 | -0.146 | -0.146 | -0.131 |
|  | (0.138) | (0.135) | (0.167) | (0.137) |
| domainAgriculture | -0.551*** | -0.551*** | -0.551*** |  |
|  | (0.162) | (0.161) | (0.032) |  |
| domainScience | 0.026 | 0.026 | 0.026 |  |
|  | (0.152) | (0.147) | (0.059) |  |
| domainLife | 1.047*** | 1.047*** | 1.047*** |  |
|  | (0.141) | (0.142) | (0.065) |  |
| domainSecurity | 0.135 | 0.135 | 0.135* |  |
|  | (0.284) | (0.313) | (0.068) |  |
| domainNatural_rm | 0.534*** | 0.534*** | 0.534*** |  |
|  | (0.120) | (0.117) | (0.035) |  |
| domainEnergy | 0.096 | 0.096 | 0.096+ |  |
|  | (0.179) | (0.177) | (0.054) |  |
| sexFemale | -0.197* | -0.197* | -0.197* | -0.188* |
|  | (0.086) | (0.087) | (0.076) | (0.086) |
| regionАстана | -0.186+ | -0.186+ | -0.186 |  |
|  | (0.107) | (0.109) | (0.124) |  |
| regionШымкент | -0.363 | -0.363+ | -0.363* |  |
|  | (0.223) | (0.219) | (0.175) |  |
| regionOther | -0.282* | -0.282* | -0.282* |  |
|  | (0.133) | (0.134) | (0.124) |  |
| org_prestigeNational | -0.295+ | -0.295+ | -0.295 | -0.185 |
|  | (0.153) | (0.155) | (0.239) | (0.154) |
| org_prestigeInternational | 0.321 | 0.321 | 0.321 | 0.350 |
|  | (0.390) | (0.385) | (0.382) | (0.390) |
| org_prestigeOther | 0.073 | 0.073 | 0.073 | 0.170 |
|  | (0.127) | (0.128) | (0.127) | (0.128) |
| pr_ranksecond | -0.726*** | -0.726*** | -0.726*** | -0.723*** |
|  | (0.145) | (0.153) | (0.216) | (0.145) |
| pr_rankbest | -0.102 | -0.102 | -0.102 | -0.101 |
|  | (0.112) | (0.115) | (0.122) | (0.112) |
| pr_ranktie | 0.408 | 0.408 | 0.408 | 0.386 |
|  | (0.477) | (0.430) | (0.565) | (0.476) |
| inst_capWorks with | 0.590*** | 0.590*** | 0.590** | 0.579*** |
|  | (0.102) | (0.106) | (0.186) | (0.102) |
| inst_capMember | 3.088*** | 3.088*** | 3.088*** | 3.069*** |
|  | (0.337) | (0.274) | (0.270) | (0.335) |
| inst_cap(Missing) | 0.279 | 0.279 | 0.279 | 0.238 |
|  | (0.407) | (0.401) | (0.357) | (0.407) |
| SD (Intercept region) |  |  |  | 0.095 |
| SD (Intercept domain) |  |  |  | 0.454 |
| Num.Obs. | 4488 | 4488 | 4488 | 4488 |
| AIC | 3910.0 |  |  | 3927.4 |
