# Supplementary material for: Negotiating science funding: The interplay of merit, bias, and administrative discretion in grant allocation in Kazakhstan
Source: PLoS One. 2025 May 30;20(5):e0318875. doi: 10.1371/journal.pone.0318875 (PMC12124552; doi:10.1371/journal.pone.0318875)
Supplement: S5 Table — This table compares the full logistic model with two lasso regressions (at different levels of lambda). The levels of lambda are chosen based on glmnet internal criteria. Made in glmnet R package [49, 52]. (DOCX) [file pone.0318875.s005.docx]

| term | estimate | min_estimate | 1se_estimate | min_lambda | 1se_lamda |
| --- | --- | --- | --- | --- | --- |
| (Intercept) | -7.5538 | 7.4787 | 6.7810 | 9e-04 | 0.0121 |
| score | 0.2324 | -0.2295 | -0.2062 | 9e-04 | 0.0121 |
| hirsh | 0.0185 | -0.0196 | -0.0189 | 9e-04 | 0.0121 |
| rintsYes | 0.1206 | -0.1094 | 0.0000 | 9e-04 | 0.0121 |
| scopusYes | 0.0967 | -0.0767 | 0.0000 | 9e-04 | 0.0121 |
| delistedYes | -0.0466 | 0.0365 | 0.0000 | 9e-04 | 0.0121 |
| win_2014Yes | 0.5161 | -0.5021 | -0.3635 | 9e-04 | 0.0121 |
| degreeDoctor | 0.4344 | -0.4254 | -0.3258 | 9e-04 | 0.0121 |
| degreePhD | -0.1455 | 0.1180 | 0.0000 | 9e-04 | 0.0121 |
| domainAgriculture | -0.5509 | 0.5522 | 0.3659 | 9e-04 | 0.0121 |
| domainScience | 0.0257 | 0.0000 | 0.0000 | 9e-04 | 0.0121 |
| domainLife | 1.0465 | -0.9916 | -0.6509 | 9e-04 | 0.0121 |
| domainSecurity | 0.1349 | -0.0598 | 0.0000 | 9e-04 | 0.0121 |
| domainNatural_rm | 0.5342 | -0.4921 | -0.2503 | 9e-04 | 0.0121 |
| domainEnergy | 0.0958 | -0.0490 | 0.0000 | 9e-04 | 0.0121 |
| sexFemale | -0.1966 | 0.1926 | 0.0741 | 9e-04 | 0.0121 |
| regionАстана | -0.1860 | 0.1557 | 0.0000 | 9e-04 | 0.0121 |
| regionШымкент | -0.3628 | 0.2879 | 0.0000 | 9e-04 | 0.0121 |
| regionOther | -0.2819 | 0.2323 | 0.0000 | 9e-04 | 0.0121 |
| org_prestigeNational | -0.2948 | 0.2263 | 0.0000 | 9e-04 | 0.0121 |
| org_prestigeInternational | 0.3211 | -0.2687 | 0.0000 | 9e-04 | 0.0121 |
| org_prestigeOther | 0.0729 | -0.1058 | -0.1169 | 9e-04 | 0.0121 |
| pr_ranksecond | -0.7263 | 0.6865 | 0.3009 | 9e-04 | 0.0121 |
| pr_rankbest | -0.1022 | 0.0735 | 0.0000 | 9e-04 | 0.0121 |
| pr_ranktie | 0.4077 | -0.3335 | 0.0000 | 9e-04 | 0.0121 |
| inst_capWorks with | 0.5900 | -0.5522 | -0.2790 | 9e-04 | 0.0121 |
| inst_capMember | 3.0876 | -2.9971 | -2.1811 | 9e-04 | 0.0121 |
| inst_cap(Missing) | 0.2790 | -0.1765 | 0.0000 | 9e-04 | 0.0121 |
