# Supplementary material for: Negotiating science funding: The interplay of merit, bias, and administrative discretion in grant allocation in Kazakhstan
Source: PLoS One. 2025 May 30;20(5):e0318875. doi: 10.1371/journal.pone.0318875 (PMC12124552; doi:10.1371/journal.pone.0318875)
Supplement: S1 Fig — The dependent variable is Score, all the available explanatory variables are used. (PDF) [file pone.0318875.s006.pdf]

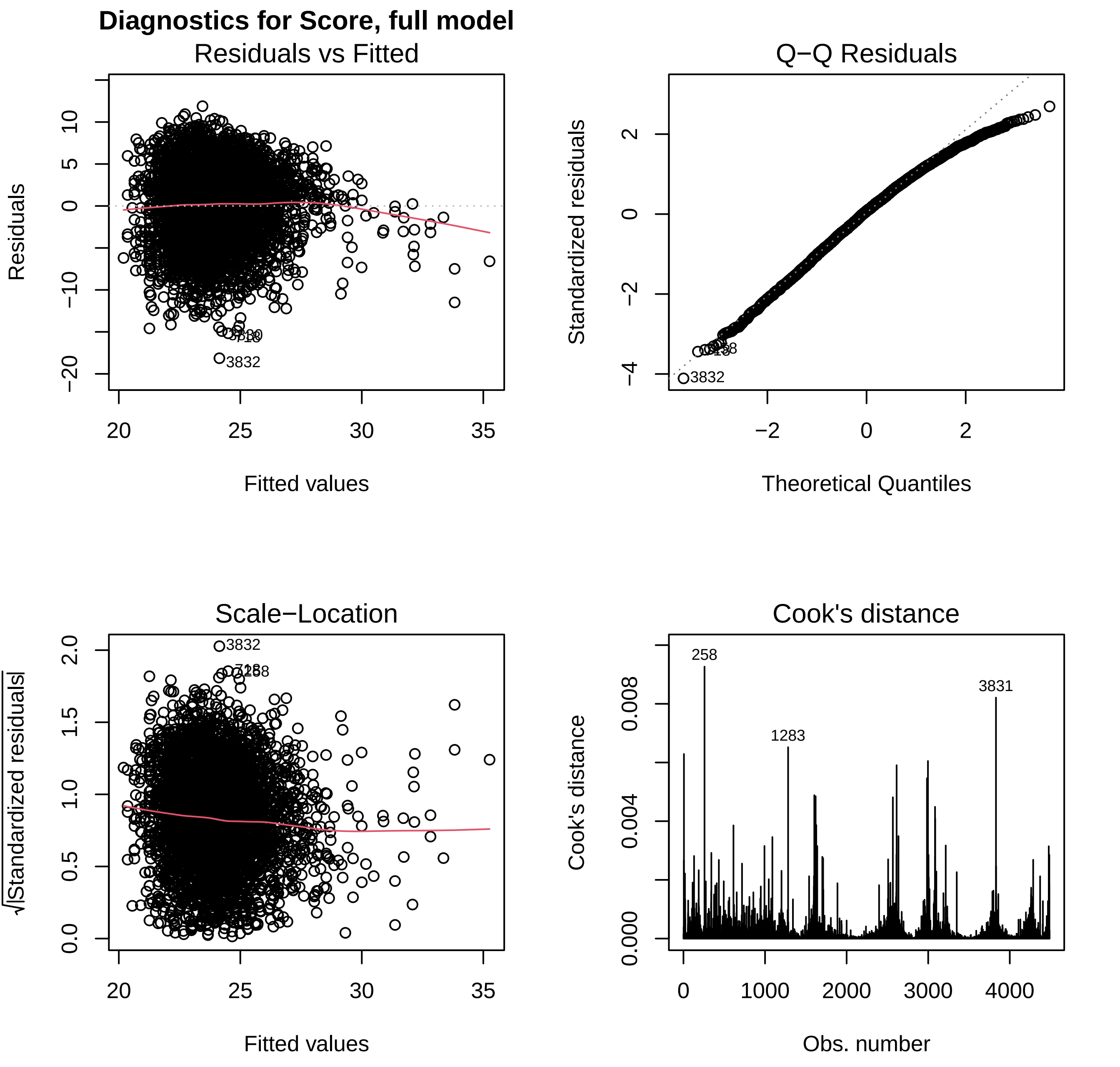
Diagnostics for Score, full model
Residuals vs Fitted
Residuals
Fitted values
3832
Q−Q Residuals
Standardized residuals
Theoretical Quantiles
3832
Scale−Location
√|Standardized residuals|
Fitted values
3832
Cook's distance
Cook's distance
Obs. number
258
1283
3831
