# Supplementary figures and images for: Negotiating science funding: The interplay of merit, bias, and administrative discretion in grant allocation in Kazakhstan
Source: PLoS One. 2025 May 30;20(5):e0318875. doi: 10.1371/journal.pone.0318875 (PMC12124552; doi:10.1371/journal.pone.0318875)

Misclassification Error

27 27 26 26 25 23 21 15 13 12 12 9 7 6 2 1 1 1

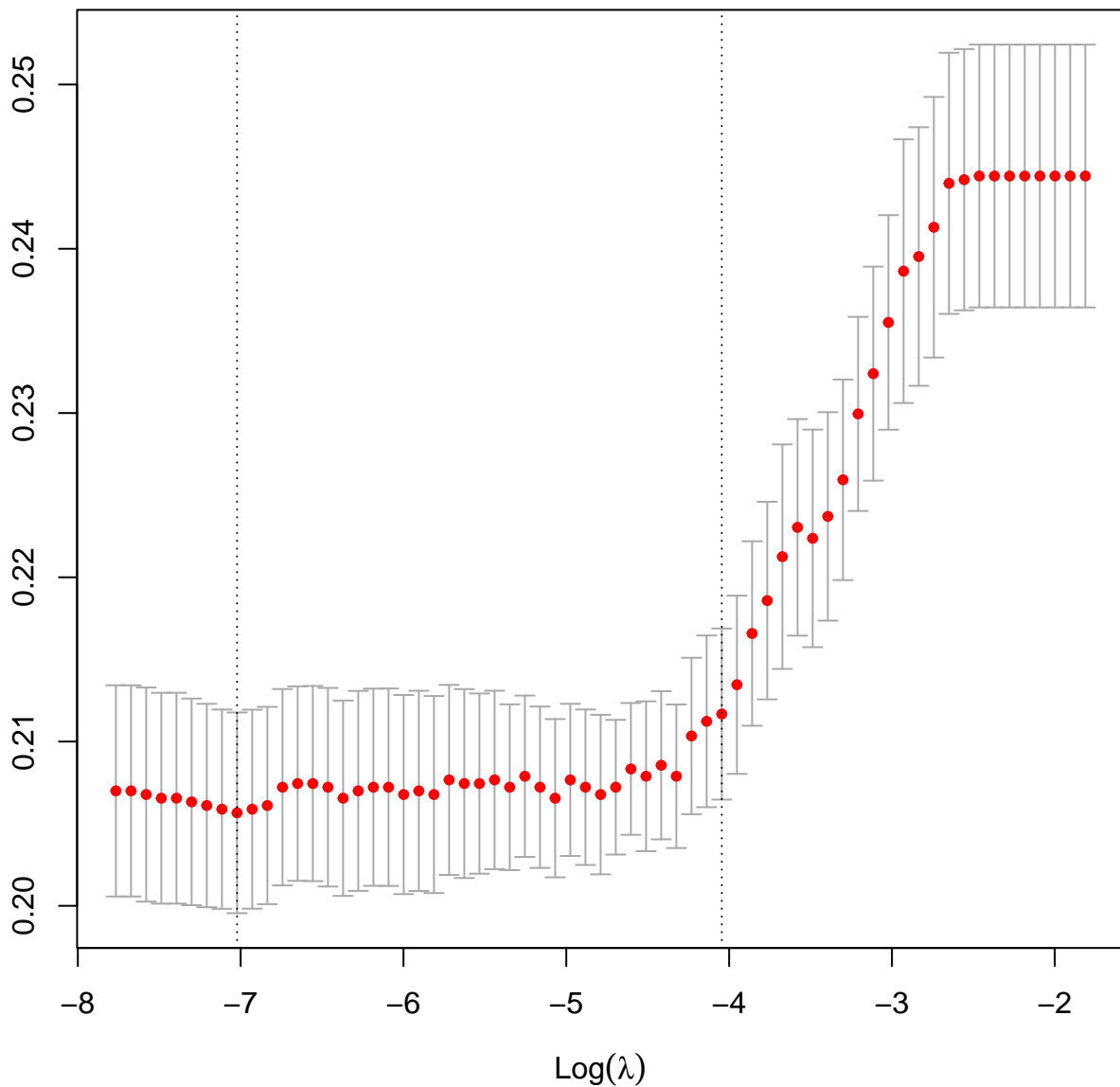

Supplement: S2 Fig — GLMNET’s selected lambda values. Made in glmnet R package [49, 52]. (PDF) [file pone.0318875.s007.pdf]

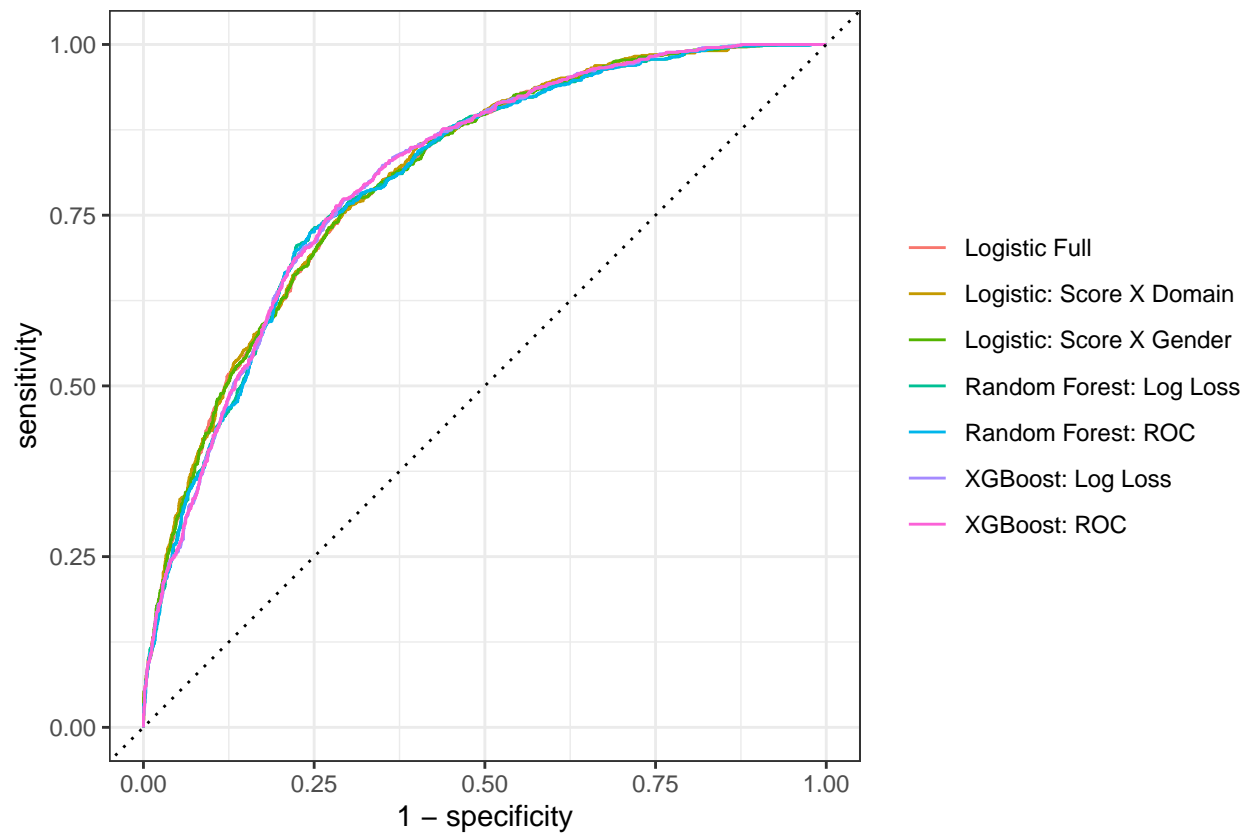

Supplement: S3 Fig — The dependent variable is winning a grant. The diagram shows overall accuracy of the both parametric and non-parametric models. (PDF) [file pone.0318875.s008.pdf]

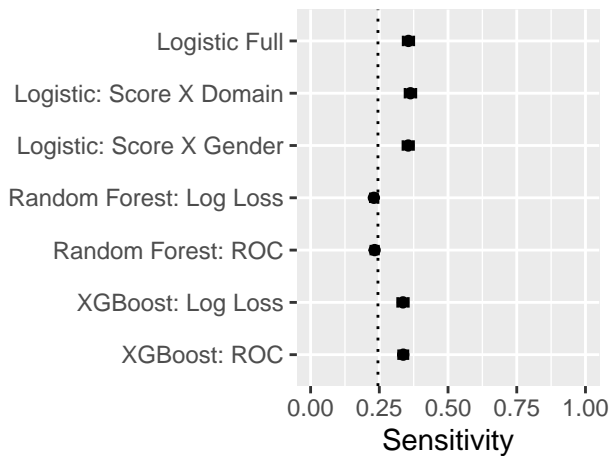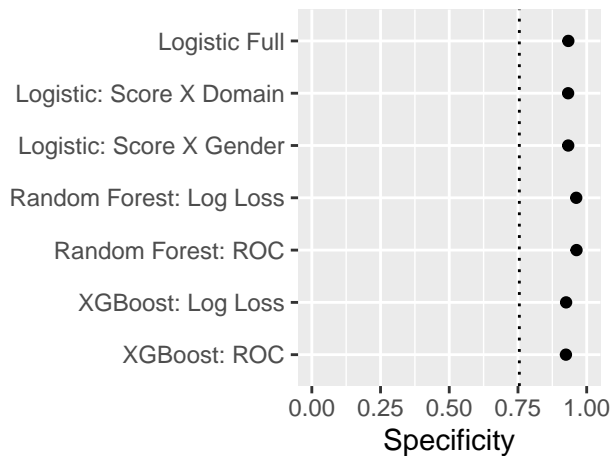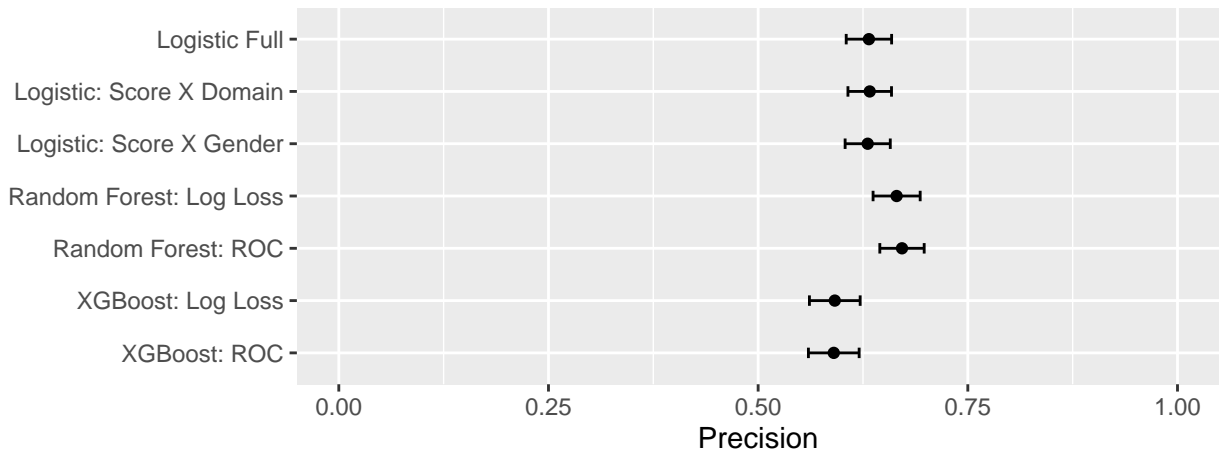

Supplement: S4 Fig — Logistic regressions tend to detect winning projects more often and are more sensitive than non-parametric models (fewer type II errors). The random forest predicts negative cases better because it is more conservative. For example, it predicts winning a grant only for 11% of applications. However, it does so, its more precise than other models. Logit regression and XGBoost tend to be more optimistic (they predict success for 14% of applications). All of these predicted success rates are still below the actual one (25%). (PDF) [file pone.0318875.s009.pdf]
