# Supplementary material for: Negotiating science funding: The interplay of merit, bias, and administrative discretion in grant allocation in Kazakhstan
Source: PLoS One. 2025 May 30;20(5):e0318875. doi: 10.1371/journal.pone.0318875 (PMC12124552; doi:10.1371/journal.pone.0318875)
Supplement: Attachment — Submitted filename: Ref_PONE-D-24-32179.pdf [file pone.0318875.s010.pdf]

Referee Report

Negotiating science funding: The interplay of merit, bias, and administrative discretion in grant allocation in Kazakhstan

Ref. PONE-D-24-32179

The paper analyses the mechanisms of selection of public funding for research projects in different fields of analysis in Kazakhstan. the study contributes to the recent research evaluation literature by proposing a different perspective from most published analyses that focus mainly on Anglo-Saxon and/or European countries. The empirical analysis of the grant funding competition held in 2017 in Kazakhstan highlights the subjective nature of the selected research projects' quality concept that unmasks selection mechanisms based on homophily and gender discrimination.

The econometric analysis is rich and interesting; however, the article needs more effort in outlining conclusions that can provide policy recommendations from the results obtained so that an improvement in public research funding selection processes can be proposed. Which selection mechanisms could correct the anomalies found? For instance, in order to correct possible gender biases in the evaluation, some institutions have proposed a quota system with an ex-ante fixed percentage of funded projects for IPs belonging to the minority sex. Could this be a feasible way forward for the analysed context? To make the paper suitable for publication I would recommend expanding and supplementing the conclusions with policy recommendations.

Furthermore, concerning the literature review, I recommend supplementing the background section with literature about homophily in research, such as:

Santos, J.M., Horta, H. & Feng, S. Homophily and its effects on collaborations and repeated collaborations: a study across scientific fields. Scientometrics 129, 1801-1823 (2024). https://doi.org/10.1007/s11192-024-04950-3

Zhou, S., Chai, S. Richard & Freeman, B. Gender homophily: In-group citation preferences and the gender disadvantage, Research Policy, 53 (1): 104895 (2024), ISSN 0048-7333,

Regarding the econometric analysis:

- The variable PI's sex is described as ‘male is the reference category’, however in the tables, the variable is defined as ‘female’ implying that instead, female is the reference category

- To complete the gender analysis, I would propose to include interaction terms in the logit model, for example, female#score and female#Hindex

- In the current version, the cross-validation section does not add any insight into the analysed phenomenon. I would suggest moving the section to the appendix or otherwise better explain how the results of the random forest and XGBoost models can complement the results obtained with the parametric methods in the previous sections.

I hope that these comments will be useful for authors.
