# Supplementary material for: Negotiating science funding: The interplay of merit, bias, and administrative discretion in grant allocation in Kazakhstan
Source: PLoS One. 2025 May 30;20(5):e0318875. doi: 10.1371/journal.pone.0318875 (PMC12124552; doi:10.1371/journal.pone.0318875)
Supplement: Attachment — Submitted filename: Response to Reviewers.docx [file pone.0318875.s011.docx]

Thank you very much for the thorough and valuable feedback, as well as for the opportunity to revise and resubmit our manuscript to *PLoS ONE*. We have divided our response into three parts: (1) comments from the Editors (2) comments from Reviewer 1, and (3) comments from Reviewer 2.

Response to the Editor

Dear Prof. Baccini,

Thank you very much for your valuable feedback.

We made every effort to carefully respond the reviewers' comments and address all their questions thoroughly. The argument has been streamlined, and the article's contributions have been strengthened. The conclusion has been thoroughly rewritten and expanded to include detailed recommendations.

Additionally, we closely adhered to the journal's requirements. We included the statement, *"The funders had no role in study design, data collection and analysis, the decision to publish, or the preparation of the manuscript,"* in the financial disclosure section. The data have been anonymized and uploaded to a public repository in compliance with the journal's guidelines. The manuscript has been reviewed for style according to PLOS ONE's requirements, and we have made every effort to correct grammatical mistakes and inconsistencies.

Response to Reviewer 1

We thank Reviewer 1 (R1) for their extremely helpful comments and suggestions. The feedback helped us to strengthened and clarify the paper's arguments.

Let us move on to specific comments.

R1 suggested enhancing the conclusion of our article by incorporating policy recommendations based on the received results. These recommendations could prove valuable in improving the selection processes. Specifically, R1 wrote:

*however, the article needs more effort in outlining conclusions that can provide policy recommendations from the results obtained so that an improvement in public research funding selection processes can be proposed. Which selection mechanisms could correct the anomalies found? For instance, in order to correct possible gender biases in the evaluation, some institutions have proposed a quota system with an ex-ante fixed percentage of funded projects for IPs belonging to the minority sex. Could this be a feasible way forward for the analysed context? To make the paper suitable for publication I would recommend expanding and supplementing the conclusions with policy recommendations.*

We have added the following passage to the conclusion section (lines 641-670),

*Based on the findings, several policy recommendations can be made to improve the efficiency of grant allocation. The first recommendation addresses gender inequality. It is crucial to determine whether this inequality is a recurring issue in grant competitions or specific to certain calls or domains. As our data suggest gender bias in the 2017 call was more pronounced in the domain of Natural Resource Management, mostly composed of projects related to oil industry.*

*If gender inequality proves to be a consistent problem across domains, implementing temporary quotas could help make up for biases. Previous research has shown that, while Kazakhstani academia has achieved quantitative gender parity — evidenced by the growing number of women obtaining scientific degrees — significant gender inequality persists [49]. In this context, such measures could be highly effective.*

*The second key policy recommendation concerns the members of the National Scientific Council (NSC). As decision-makers, they were also involved in the competition, which created a clear conflict of interest. The Ministry of Science and Higher Education has already taken steps to address this issue. In subsequent competitions, NSC members no longer have the final say in determining which projects receive funding; instead, they score projects alongside external reviewers. However, further in-depth research is needed to clarify the councils role, evaluate their contributions to the final decisions, and open the "black box" of communication between council members during the negotiations. For example, one mechanism that may contribute to gender bias is gender homophily coupled with disproportionate number of men in research councils. In other words, possible interventions may focus not on the outcome of the competition ( for example, gender quotas for winning projects), but on gender composition of the councils (gender quotas for council members).*

*It would be misguided to assume that the issue with research councils is unique to Kazakhstan. Similar cases have been observed in European contexts, where selection committees sometimes disregard review scores [6] or exhibit self-serving bias [41]. Therefore, it is important not to exoticize the Kazakhstani case but to develop a unifying framework for addressing issues of gender inequality and the balance between external and internal expertise.*

R1 recommended supplementing the background section with literature about homophily in research. In particular, he/she suggested the following sources of literature:

*Santos, J.M., Horta, H. & Feng, S. Homophily and its effects on collaborations and repeated collaborations: a study across scientific fields. Scientometrics 129, 1801-1823 (2024). https://doi.org/10.1007/s11192-024-04950-3*

*Zhou, S., Chai, S. Richard & Freeman, B. Gender homophily: In-group citation preferences and the gender disadvantage, Research Policy, 53 (1): 104895 (2024), ISSN 0048-7333*

Thank you very much for these useful sources of literature. We have updated the manuscript by quoting these sources in the theory section.

We have augmented Background and Theory section with the following text (p. 4, lines 122-134):

*Finally, it is crucial to recognize that non-meritocratic factors, such as an applicant's gender, ethnicity, or race, can significantly impact the decision-making process. For example, gender often serves as a primary characteristic for homophily in scientific collaborations, meaning that individuals tend to form partnerships and networks with those of the same gender. As a consequence, not only collaboration networks but also citation networks show systematic gender homophily [33; 34]. Research also shows systematic penalties for women in both hiring and grant funding [4; 8].*

We also cited these research when describing our independent variable - PI’s gender.

*The variable PI's sex is described as ‘male is the reference category’, however in the tables, the variable is defined as ‘female’ implying that instead, female is the reference category*

We apologize for the confusion. In the regression tables, we write “Female PI” to indicate that it is a coefficient reflecting female PI’s chances relative to the reference category - “male PI”. To avoid confusion, we have added clarifications in the captions.

*To complete the gender analysis, I would propose to include interaction terms in the logit model, for example, female#score and female#Hindex*

We have incorporated interaction terms in the logit models in the table 4 (page 16)

*In the current version, the cross-validation section does not add any insight into the analysed phenomenon. I would suggest moving the section to the appendix or otherwise better explain how the results of the random forest and XGBoost models can complement the results obtained with the parametric methods in the previous sections.*

We agree that in its current form cross-validation section does not add much insight. Our aim, however, was to illustrate underperformance of more flexible non-parametric models. Yet again, we agree that cross-validation has minimal impact on the main findings; therefore, we have relocated this analysis to the supplementary materials.

We appreciate R1’s thoughtful remarks on our manuscript and helpful suggestions.

**Response to Reviewer 2**

We are grateful to Reviewer 2 (R2) for immensely useful comments and feedback on our paper.

R2 provided three main comments, which we will address first. The first comment pertains to a reflection on the role of reviewer scores. R2 writes,

“Beginning with the first point on reviewer scores. As is pointed out in the front-end of the paper, oftentimes a single negative review is all it takes for a grant to be declined. In general, any reviewer disagreement may result in rejection: seeing discordant opinions, whomever takes the final funding decisions (e.g. the panel) may simply decide that the project is too risky or controversial, and reject it (see e.g. Lane et al. 2022, <https://doi.org/10.1287/mnsc.2021.4107> ). I think this might be of consequence for how review scores are operationalized in the analyses. Let me make an example to illustrate my point: two proposals are competing for the same grant and have been reviewed by the same set of reviewers. Proposal A received the scores {7, 7, 7, 7}. Proposal B has received the scores {8, 8, 8, 4}. Both proposals have the same average score of 7: this means that, if we use the average score as predictor, we would expect A and B to have the same chances of being funded. However, reviewers are in consensus for proposal A, and in (strong) disagreement for proposal B. What happened is that one of the reviewers of B may have identified problems that the other reviewers have not noticed. The panel who is tasked with the final decision might think that proposal B is therefore riskier than proposal A, and ultimately choose B over A. This goes to show that reviewer agreement, according to theory, may be as good a predictor of funding success as the average score. Hence my question: have you considered estimating the models using some measure of disagreement (e.g. the standard deviation of scores) as fixed effect in place of or in addition to the average score? Do the results say anything new?”

This is certainly a very important commentary that highlights the issue of reviewer agreement. We would be glad to test this idea; however, the data are structured in a way that makes it impossible. Specifically, we only have access to the average rating from three reviewers, not the individual ratings. During the data collection phase, we attempted to request data on each individual reviewer’s scores, but our request was denied. For reasons that remain unclear, these scores are considered classified information. Consequently, only the average review score could be used in this study.

R2's second main comment was about data sharing statement, they wrote

“My second main comment is about the data stamen that “Data cannot be shared publicly because it contains PI's full names”. And then “The data

underlying the results presented in the study are available from

[email] upon request”. Then is the data available or not available for secondary scrutiny? If it is available, it should be published to a persistent repository and made Findable, Accessible, Interoperable and Reusable (FAIR). If this is not possible, then how could it be made available “upon request”? Note also that, if the identifiability of PIs is the only obstacle to publishing the data, perhaps PI names can be replaced with anonymized IDs.”

We have anonymized our dataset and uploaded it to a public repository.

R2's third main comment concerns a complaint about the use of judgmental language.

First, we apologize for the careless choice of words. Most of the authors are sociologists themselves, and we may have become overly critical in our self-reflection. We did not mean to devalue the work of fellow sociologists. We have rewritten the related paragraphs in a more professional and neutral tone. And we appreciate the restatement suggested by the reviewer.

In particular, we have either completely deleted or rewritten paragraphs in lines 78-87 which reads now,

*Partly, peer reviews can appear inconsistent due to factors such as reviewer fatigue, vague evaluation criteria (e.g., significance or novelty), or a lack of reviewing experience [14, 24]. However, disagreements between reviewers also stem from the inherent ambiguity of merit. Scientific communities may agree on what constitutes quality science and who among them comes closest to doing it, yet the literature indicates that consensus is not always reached. Many scientific fields have controversial topics that split communities into what are often called schools of thought [12, 25]. While the degree of (perceived) fragmentation vary across disciplines [25–27], sociological studies of scientific knowledge show that the nature of scientific fact can be, and often is, contested even in the most stable of hard sciences like physics or chemistry [28–31].*

We have also deleted a footnote on the page 4;

and we have rewritten the paragraph in the lines 179-185 (now it is lines 172-183), which now reads,

*Scientific disciplines vary in the extent to which they agree on preferred endeavors, theories, methods, or tools. Some disciplines feature institutional structures that impose a higher degree of homogeneity on the education and training required for students to begin their scientific careers [37, 38]. Others disciplines exhibit greater diversity in their epistemic styles [2]. We argue that the level of epistemic diversity may correlate with the stability of evaluation criteria. Specifically, disciplines with more homogenous epistemic styles may tend to have more fixed sets of criteria for distributing resources (e.g., grants, academic positions). This suggests that in fields with low epistemic diversity, science managers may be more inclined to rely on external experts, whose authority helps reduce uncertainty (and potentially shift responsibility). Conversely, disciplines characterized by more diverse epistemic cultures often, by having more flexible evaluation criteria, allow administrators to exercise greater discretion in their decisions.*

Reviewer 2 also writes,

The description of the evaluation scales on page 7 explains that the criterial scores are graded on a scale from 0 to 9. Since the direction of this scale is directly relevant for interpreting the results, the text should also explicitly say whether higher values represent better score (thus, 9 is the best grade) or the other way round (0 as the best grade).

We agree and we have explicated the direction of the scale in the text, now it reads (lines 308-310),

*each application had been graded by three independent anonymous experts based on four criteria. Each criteria ran on 0 to 9 scale, where 9 is the best grade, and consisted of 2-3 categories with a set of cue questions.*

Reviewer 2 writes,

about the variable “PI’s formal degree”. Based on your description, It seems to me that this variable captures a cohort effect rather than simply the amount of accumulated “social capital”, as you put it. Younger academics (i.e. those with a PhD), I would expect, are at a disadvantage because of a variety of effects – they have had fewer chances to accumulate previous funding; they have had less time to accumulate good and well-cited publications; and sure, they would have had less time to form those social ties that may favor them in a national competition (social capital). Separating younger and older academics there are also institutional changes that only the older generation has lived through: this might have had an impact on how they work and what they decide to work on. These and other effects can all be lumped together under the concept of “cohort effect”. If you agree, please revise the name and description of this variable.

We agree that a cohort effect would be a better description; we have revised the the corresponding paragraph which now reads (lines 374-382),

*PI’s formal degree (categorical, 3 levels). This variable has three categories: Candidate of Science (reference level), Doctor of Science, and PhD. Before joining the Bologna Process in 2011, Kazakhstan followed the Soviet two-tier system of scientific degrees: Candidate of Science (first tier) and Doctor of Science (second tier). Since the 2000s, this system has been replaced by the single PhD degree. Because we do not have data on the year of degree award, this variable may capture cohort effects across different generations of academics. It likely reflects variation in the accumulation of social capital throughout participants’ careers, as well as differences in institutional experience, both of which may influence the likelihood of funding.*

Reviewer 2 writes,

about the variable “previous success”: this encodes whether the PI has won a call in the previous call in 2014. Note that PI might have acquired funding from other calls or other funders as well. If you agree, my advice is to make it explicit that this variable will have some (or even many?) false negatives, meaning that some/many PIs will be coded as “previous success = FALSE” even though they had previous success in calls other than the one in 2014. You may try to claim that this is a conservative estimate of previous success that seeks to capture exactly what you are interested in – previous success but also previous familiarity with what you call the “rules of the game” in science funding in Kazakhstan.

We fully agree with this observation. Indeed, a researcher's experience may extend far beyond winning grant competitions held only by the Ministry. In line with the reviewer's recommendations, we have updated the description of this variable. We noted the lack of data on the applicant's full grant track record and emphasized the conservatism of our assessment. Now the paragraph reads (lines 365-373),

*this variable indicates whether a PI received funding in the previous grant competition held in 2014. Although winning the previous competition likely correlates with a PI’s academic fitness, this variable can also approximate a PI’s experience with the selection process or signal "grant-worthiness" to the panelists in the absence of other cues. Ideally, we would like to have information on the full grant track record of participants, including all types of funding they received. In the absence of such data, we interpret this variable as a conservative indicator of previous experience, as it only captures those who participated in the previous round of the grant competition (thus increasing the rate of false negatives).*

Reviewer 2 writes,

The description of the variable “PI’s sex” uses interchangeably gender (man, woman) and sex (male, female). My general advice is to choose one of the two consistently and to name this variable accordingly. The standard, to my understanding, is to use gender: hence my recommendation to choose gender if applicable. Please check if PlosOne has specific author guidelines for talking about sex, gender, and inclusive language. On the other hand, I can imagine good reasons for using sex (male/female) instead. For example, applicant forms might have explicitly asked for the biological sex of applicants rather than their gender: in this case it would make sense to use the same categories as they appear on the form, and to add a footnote or a comment to this effect. I am not familiar with the funding landscape of Kazakhstan and I cannot speak Kazakh or Russian, so I will leave it for you to determine whether this is the case.

We are grateful to the reviewer for pointing this out. We have systematized the usage to “gender” and added a footnote explaining uncertainty associated with this category.

In particular, now the text reads (lines 434-462):

*PI’s gender (binary, men is the reference category). We use the term gender not in the sense of self-identification but rather to describe how individuals were categorized within the register system used to collect applications. We acknowledge that the use of gender instead of sex is debatable, as the system apparently referred to the sex assigned on identification documents (e.g., national passports). Our argument for using gender, however, is that it was likely the panelists’ and reviewers’ perception of applicants’ sex that influenced their decisions. In that sense, since this perception was socially conditioned and tied to the identification system imposed by state institutions, we assume that gender is a more appropriate term.*

Table 1 provides descriptive statistics for some of the variables. I would be advisable to show the distribution of the other variables as well, possibly in an appendix.

We have added a summary table and figures in the supplementary materials.

*Minor comments:*

What I miss form the conclusion/discussion is a comment on the potential (?) generalizability of these results to other contexts. For example, knowing little about the subject I would naively think that other post-Soviet institutions have undergone similar policy changes and social processes. Do we know of – or should we expect – similar dynamics at play in other post-soviet states?

In response to this comment, we have expanded our conclusion by incorporating recommendations, offering a brief comparison of our study with findings that made by other scholars, and outlining prospects for future research in lines

Gender bias in science funding is quite controversial. Several studies report gender differences in funding success and grant sizes (Bornmann, Mutz, and Daniel 2007 https://doi.org/10.1016/j.joi.2007.03.001 ; McAllister, Juillerat and Hunter 2016 https://doi.org/10.1038/529466d ; Burns et al. 2019 https://doi.org/10.1371/journal.pmed.1002935 ; Witteman et al. 2019 https://doi.org/10.1016/S0140-6736(18)32611-4 ). However, a couple of other studies find the opposite (Mutz, Bornmann and Daniel 2012 https://doi.org/10.1027/2151-2604/a000103 ; Yip et al. 2020 https://doi.org/10.1057/s41599-020-00656-y ). Perhaps you could explicitly link your results to this stream of literature by showing yet another domain where there is evidence for a gender effect.

We are very gratefull for the list of publications, we have included a short summary on it which reads (lines 443-457),

*A PI’s gender is an important variable to account for, as research documents controversial evidence of gender biases in academia. For example, McAlister and colleagues report that "Women make up 33% of the applicants who are eligible for programmes funded by the UK Biotechnology and Biological Sciences Research Council, but they lead only 21% of grant applications" [43]; Burns and colleagues find that women had significantly lower success rate in applications submitted to the Canadian Institutes of Health Research [44]. Using a natural experiment data from the same institution, Witteman and colleagues also document that women success rate was lower than that of men by a significant margin, age and research domain adjusted [45]. On the other hand, a study of the Australian Science Fund program find no gender gap in review scores [20] and a study of grant in the Social Science department at the University of Hong Kong find even slight women advantage in grant award success rates [46]. This mixed evidence, indeed, suggests that contexts matters, and examining the case of Kazakhstan may contribute towards building a processual and situational understanding of gender in academia.*

The first paragraph of the introduction can be sharpened in a couple of places where the current phrasing is not quite precise. One issue is with the sentence “While merit is often cited as the ideal criterion […]”: cited by whom? Also note that there other criteria that some funders and scholars may deem to be even more important than merit, such as scientific relevance or the potential for impact. An easy workaround here could be to either provide some references or to tone down this statement by rewriting it along these lines: “Merit is certainly an important criterion; however, […]”.

The other sentence in the introduction that can be sharpened is the one that reads “since science depends on public money, […]”. The issue is that not all science depends (exclusively or at all) on public money. Here, too, the fix could be quite straightforward: “since science often relies on public funds, […]”.

We agree with this feedback and we have included the reviewer’s suggestions in the introduction, the revised paragraphs now reads (lines 2-10),

*Public research grants are among the most institutionalized methods of investing in science [1], but determining who deserves funding is a complex task. While merit is certainly an important criterion [2], economic, social, and political concerns also influence funding decisions. Scientists often value autonomy and believe that their internal rankings are the best predictors of future scientific and societal impact [2, 3]. However, because science frequently relies on public funds, external actors (e.g., funding bodies, science administrators) introduce their own perspectives and agendas. The ways in which scientists and administrators negotiate and reconcile these demands present an intriguing sociological question.*

Information on the two stages of review presented across the second and third paragraph comes across as somewhat redundant. Please consider merging the two explanations.

We agree and we have merged the two paragraphs into one, now it reads (lines 11-17)

*A typical grant competition consists of two stages: first, grant applications are reviewed and scored by subject matter experts; second, selection panels decide whether a project deserves funding. Research identifies many factors influencing positive reviews or grant awards, including scientific merit, evaluative cultures, administrative discretion, previous success, and, unfortunately, gender and race biases [2, 4–9]. However, fewer studies have explicitly addressed the direct interplay between review scores and funding decisions [4, 5]*

Top of the background and theory section: “scientific peers are the most suitable juries because their internal rankings predict future scientific output the best”. This is presented as a fact – if it is, it begs for supporting references. If it is an opinion, as I think it is, it should be phrased more carefully.

Thank you for pointing this out. We have qualified the statement. Now it reads (lines 56-58),

*“From this perspective, scientists tend to see their peers as suitable juries because*

*internal rankings within a discipline are considered a reliable measure of future scientific output.”*

The bottom of the “Context” section describes the fallout and public scandal that followed the publication of winners’ list the 2017 call. Some of it – the description of an open letter by scientists, the involvement of a former President and their ministry – is not supported by sources as I would expect (e.g. references to the open letter and newspaper articles outlining what is described).

We agree and we have added footnotes with hyperlinks leading to related newspaper articles on page 7.

Line 417: “a PI could lead at least two projects”. I presume this is an error, and the text should actually read “a PI could lead *at most* two projects”.

Thank you for pointing out, we have corrected this part.

Regression tables or their caption should remind the reader what the dependent variable is.

We have added captions with the dependent variable for each regression table.

Once again, we wish to express our gratitude to R2 for sharing their perceptive thoughts and comments.
