# Supplementary material for: Negotiating science funding: The interplay of merit, bias, and administrative discretion in grant allocation in Kazakhstan
Source: PLoS One. 2025 May 30;20(5):e0318875. doi: 10.1371/journal.pone.0318875 (PMC12124552; doi:10.1371/journal.pone.0318875)
Supplement: Attachment — Submitted filename: Response to Reviewers 2.docx [file pone.0318875.s012.docx]

We thank PloS ONE editorial team as well as the reviewers for their valuable feedback and the opportunity to refine our manuscript. Below are our responses to the reviewers’ comments.

Response to Reviewer 2.

We cordially thank Reviewer 2 for their previous comments which really helped us to revise the manuscript. We also deeply appreciate their approval of our work.

Response to Reviewer 3.

We are very grateful to Reviwer 3 for their detailed methodological comments. We address the comments as follows,

1. Reviewer 3’s wrote that it would be nice if we share the code used to conduct analysis.

We totally agree and have added the replication code to the repository (at <https://dataverse.harvard.edu/dataset.xhtml?persistentId=doi:10.7910/DVN/8UZ7EY>).

2. Reviewer 3’s wrote that,

Up to what I understand, the scores are discrete and as such should be treated. Here instead the outcome is considered continuous and linear regression is applied. I cast some doubts on the appropriateness of the methods. Please, provide evidence that the Gauss-Markov assumptions are met. This is crucial and mandatory to ensure the reliability of the inferential results. Indeed, I suggest to go for a generalized linear model instead, properly accounting for the discrete nature of the outcome variable.

We thank the Reviewer for pointing this out. The scores that characterize projects are averages of three peer reviewers’ evaluations on the scale from 0 to 36. They can take fractional values (e.g. 35.67), however they are discrete in the sense that they has a finite support (their fractional part can only be .00, .33, or 0.67). In particular, score variable takes on 111 (3*37) distinct values.

The Reviewer suggested that to test Markov-Gauss assumptions. We have added model diagnostic plots to the supplement (**S1_figure.pdf**). The diagnostics indicate that residuals tend to align with a normal distribution up until upper quantiles where empirical quantiles tend to be closer to the mean that the theoretical expectation. This basically reflects the natural upper boundary for the score (36 points) and that the predicted values tend to underestimate real ones as they approach the upper boundary. To account for these issues, we, run additional regression models: with clustered standard errors and a tobit-regression (with residuals having truncated normal distribution). The results are included in the supplement materials (**S2_table.docx**).

The Reviewer also suggested using generalized linear models. We agree and we have modelled score as an ordered categorical variable. We have added the results to the supplement (**S3_table.docx**).

Finally, we would like to add that we model score variable not to explain its variation but rather to provide a thick description, to illustrate how its conditional mean is associated with various characteristics of the projects. We realise that in its current edition, the manuscript does not communicate it clearly, so we have added an explicit statement on the pages.

So, to sum up how did we address the reviewers comment.

In lines 494-504, we added the following paragraph

*We treat the variable review score here as a continuous one, while in fact it is effectively discrete as it can take on only a finite number of values. More over, conceptually it is more sound to treat score as an ordinal variable, as its values reflect levels of academic fitness assessed by the reviewers (akin to IELTS or TOEFL scores). A more principled approach would be to model reivew score with an ordinal regression treating its values as ordered levels.*

*This paper, however, aims to illustrate the effect of scores on the probability of getting a grant. In other words, review score is an explanatory variable, and our OLS regressions do not aim to explain it, but rather to describe it in relation to other variables. So as not to sidetrack too much, we organize the analysis as follows. Below, in Table 2, we present the results of OLS regressions that treat review score as a continuous variable. In the supplementary materials, we run model diagnostics and alternative models that treat score as a discrete and ordinal variable.*

In supporting information, we have added models diagnostics (**S1_figure.pdf**), and additional regression tables (**S2_table.docx**, **S3_table.docx**).

3. Reviewer 3 wrote that,

The data have a clear hierarchical structure: there are repeated measurements for some PIs, there might be a judge-effect, and also the field may have an impact. Overall, what I am saying is that there might be unobserved heterogeneity at different levels of the hierarchy which may affect the inferential results. I strongly suggest to include random effects at the different levels of the hierarchy to account for such heterogeneities. Furthermore, relying on Aitkin, M. (1999). A general maximum likelihood analysis of variance components in generalized linear models. Biometrics, 55(1), 117-128 it would be interesting to consider discrete random effects leading to a finite mixture model, i.e. they allow for clustering PIs, etc. Functions are available in R (npmlreg package), Stata (gllamm), and other software.

We agree. We have run models with random effects at the level of PI, region, and domain. We have also augmented our previous models with robust standard errors clustered at the domain level. In the **tables 4 and 5** of the manuscript we fit models with robust standard errors. In the supporting information, we have added a table that compare reported tables with mixed-effects logistic regressions (**S4_table.docx**).

4. Reviewer 3 wrote that,

Budget constraints on the probability of winning may also be an issue. I have to admit that I do not the specific context, but in my country the budget is allocated field-wise to ensure that all the fields would receive some funds. Moreover, there might be the case where two projects share the same grade but one only is financed due to lack of economic resources.

We absolutely agree — it is likely that budget matters, in the sense that council members may explicitly consider the amount of money requested when deciding for or against a project. However, there is no publicly available information on the funding amounts requested by projects; nor is there information on domain-specific constraints. In the data at hand, this is perhaps an unobserved source of variation. We have added a paragraph acknowledging this concern in lines (**646-650**).

*Part of this "unexplained" success rate is likely due to unobserved project characteristics. For example, some projects might be funded simply because they were relatively inexpensive. In other words, council members might take the requested budget into account when deciding for or against a project. Unfortunately, we could not find publicly available data on project budgets.*

5. Reviewer 3 wrote

It would be nice to see how well the logistic model (again I suggest to move to a logistic mixed model instead) is able to correctly identify the funded projects. Specificity and sensitivity could be added to the analysis, for example.

We agree and we have added model performance tests (specificity and sensitivity along a set of other metrics) to the supporting information (**S3_figure.pdf**, **S4_figure.pdf**).

We have also a short cross-validaiton section (lines 624-640) that reads,

*Logistic regression is a well-established statistical method to model probability of a given event. However, in terms of predictive performance, logistic regressions do not always match with more recent non-parametric predictive models such as random forest. This predictive performance, however, comes at the cost of interpretation; non-parametric models do not have a closed-form equation connecting response and predictor variables. Nevertheless, comparing logit models with more flexible non-parametric models in terms of predictive performance helps us illustrate that in our case logistic models are on par with non-parametric ones.*

*We estimate the performance of the models using two 10-fold cross-validations. We choose three models from the logistic regression branch, one full model specification (with all available predictors), and two models with interaction effects (score X domain and score X sex). We also select two specifications for both random forest and XGBoost models. For those non-parametric models, we fine-tune parameters over a Latin hypercube grid to optimize the receiver operator area under the curve (roc auc) and logarithmic loss (log loss). S3 Figure and S4 Figure in the supplement materials summarize our performance tests. The tests were done in Tidymodels R package [50].*

6. Reviewer 3 wrote,

As a plus, I suggest to consider variable selection techniques. There are many independent variables in the model and most of them do not have any explanatory power. Thus, procedures like lasso, ridge or elastic net may help in identifying the main determinants.

We agree. We have run a set of lasso regressions and compated their estimates with those of regular logistic regressions. The results are added to the supporting information (**S2_figure.pdf**, **S5_table.pdf**). Also, we added the following explanation in the manuscript (lines 610-623)

*Before moving on, we would like to briefly address the issue of model selection. Our models yield many non-significant coefficients, yet we retain them. From a parsimony perspective, this is suboptimal, as it would make more sense to retain only those variables that possess greater explanatory power—and thus simplify the models.*

*The reason for retaining non-significant coefficients is primarily conceptual. Our explanatory variables were selected based on: (a) theoretical relevance and (b) data availability. Our main goal was to test whether these variables are associated with the probability of funding. When a variable is not (for example, the h-index), it still provides valuable information, as it arguably suggests that the review score already captures what the h-index measures.*

*However, to demonstrate the robustness of the coefficients, we fit several LASSO regressions [49] and compare their coefficients with those obtained in our final model (excluding interaction effects). The results are presented in the Supporting Information (S2 Figure, S5 Table).*
